# An Internet-Based Cognitive Behavioral Program for Adolescents With Anxiety: Pilot Randomized Controlled Trial

**DOI:** 10.2196/13356

**Published:** 2020-07-24

**Authors:** Kathleen O'Connor, Alexa Bagnell, Patrick McGrath, Lori Wozney, Ashley Radomski, Rhonda J Rosychuk, Sarah Curtis, Mona Jabbour, Eleanor Fitzpatrick, David W Johnson, Arto Ohinmaa, Anthony Joyce, Amanda Newton

**Affiliations:** 1 Glenrose Rehabilitation Hospital Edmonton, AB Canada; 2 IWK Health Centre Halifax, NS Canada; 3 Dalhousie University Halifax, NS Canada; 4 Department of Pediatrics Faculty of Medicine & Dentistry University of Alberta Edmonton, AB Canada; 5 Department of Neurosurgery University of Ottawa Ottawa, ON Canada; 6 University of Calgary Calgary, AB Canada; 7 School of Public Health University of Alberta Edmonton, AB Canada; 8 University of Alberta Edmonton, AB Canada

**Keywords:** internet, cognitive behavioral therapy, adolescents, anxiety, randomized controlled trial, pilot

## Abstract

**Background:**

Internet-based cognitive behavioral therapy (ICBT) is a treatment approach recently developed and studied to provide frontline treatment to adolescents with anxiety disorders.

**Objective:**

This study aimed to pilot procedures and obtain data on methodological processes and intervention satisfaction to determine the feasibility of a definitive randomized controlled trial (RCT) to test the effectiveness of a self-managed ICBT program, Breathe (Being Real, Easing Anxiety: Tools Helping Electronically), for adolescents with anxiety concerns.

**Methods:**

This study employed a two-arm, multisite, pilot RCT. Adolescents aged 13 to 17 years with a self-identified anxiety concern were recruited online from health care settings and school-based mental health care services across Canada between April 2014 and May 2016. We compared 8 weeks of ICBT with ad hoc telephone and email support (Breathe experimental group) to access to a static webpage listing anxiety resources (control group). The primary outcome was the change in self-reported anxiety from baseline to 8 weeks (posttreatment), which was used to determine the sample size for a definitive RCT. Secondary outcomes were recruitment and retention rates, a minimal clinically important difference (MCID) for the primary outcome, intervention acceptability and satisfaction, use of cointerventions, and health care resource use, including a cost-consequence analysis.

**Results:**

Of the 588 adolescents screened, 94 were eligible and enrolled in the study (49 adolescents were allocated to Breathe and 45 were allocated to the control group). Analysis was based on 74% (70/94) of adolescents who completed baseline measures and progressed through the study. Enrolled adolescents were, on average, 15.3 years old (SD 1.2) and female (63/70, 90%). Retention rates at 8 weeks were 28% (13/46; Breathe group) and 58% (24/43; control group). Overall, 39% (14/36) of adolescents provided feedback on completion of the Breathe program. Adolescents’ scores on a satisfaction survey indicated a moderate level of satisfaction. All but one adolescent indicated that Breathe was easy to use and they understood all the material presented. The most frequent barrier identified for program completion was difficulty in completing exposure activities. The power analysis indicated that 177 adolescents per group would be needed to detect a medium effect size (*d*=0.3) between groups in a definitive trial. Data for calculating an MCID or conducting a cost-consequence analysis were insufficient due to a low response rate at 8 weeks.

**Conclusions:**

Adolescents were moderately satisfied with Breathe. However, program adjustments will be needed to address attrition and reduce perceived barriers to completing key aspects of the program. A definitive RCT to evaluate the effectiveness of the program is feasible if protocol adjustments are made to improve recruitment and retention to ensure timely study completion and increase the completeness of the data at each outcome measurement time point.

**Trial Registration:**

ClinicalTrials.gov NCT02059226; http://clinicaltrials.gov/ct2/show/NCT02059226.

## Introduction

### Background: Anxiety, Cognitive Behavioral Therapy, and Internet-Based Approaches

Anxiety disorders are the most prevalent of mental illnesses to be diagnosed before the age of 18 years. Approximately 1 in every 3 adolescents will meet criteria for an anxiety disorder in their lifetime [1]. The global burden of such disorders in adolescence is significant. In 2010, anxiety disorders accounted for 14.6% of disability-adjusted life years (DALYs), with the highest proportion of total DALYs seen in young people aged 10 to 29 years [2].

The current classification system for diagnosing anxiety disorders identifies several types: separation anxiety disorder, selective mutism, generalized anxiety disorder (GAD), social phobia, specific phobia, panic disorder, and agoraphobia [3]. In general, these disorders are characterized by excessive, persistent fear or worry that interferes with day-to-day functioning; such impairments can be pervasive, affecting activities of daily living, school performance, and interpersonal relationships [4]. General worries are common among adolescents, and those who experience impairments that do not meet the threshold for any particular diagnosis may be considered as having a subthreshold disorder. Research exploring subthreshold anxiety among adolescents is limited; however, a 2014 study by Burstein et al [5] found that the prevalence of subthreshold GAD among adolescents in the United States was 2-fold compared with adolescents diagnosed with GAD. Eventually, subthreshold disorders may lead to a need for treatment [6].

Cognitive behavioral therapy (CBT) is a well-established first-line treatment for anxiety disorders in adolescents [4,7,8] and can reduce the risk of chronic anxiety if delivered early and effectively [9]. CBT conceptualizes anxiety as arising from maladaptive patterns of cognition and behavior, with treatment focusing on addressing the factors that maintain an adolescent’s symptoms rather than understanding the etiology of the disorder [7]. Accordingly, therapeutic content focuses on teaching skills for replacing anxious thoughts with a more realistic and adaptive approach, developing skills to cope with and reduce anxiety symptoms, and exposure to feared situations to address anxiety-driven behavior and avoidance.

Although trained mental health professionals have traditionally delivered CBT, the structured, skill-based, and sequential nature of CBT translates well to computer-based delivery. A computer-based approach to CBT delivery can involve accessing a program via the internet (internet-based CBT; ICBT), which typically involves therapeutic content being presented in web-based, structured modules in a progressive format. Technology-based features such as multimedia (eg, videos and audio files) and interactive user formats (eg, drop-down response menus) may be used to deliver therapeutic content. In some ICBT programs, therapist support may also be included in the form of messages, phone calls, or in-person contact [10].

A total of four randomized controlled trials (RCTs) provide evidence of the treatment effects of ICBT for adolescents with anxiety disorders [11-14]; two other studies with adolescent populations have been recently published, but their focus was on establishing feasibility [15,16]. The 9-session program developed by Tillfors et al [13], which represents the earliest published RCT of ICBT, targeted social fears (namely public speaking) among high school students who met diagnostic criteria for social anxiety disorder. Posttreatment, significant improvements were reported in favor of ICBT (compared with wait-list control) on measures of social anxiety (Social Phobia Screening Questionnaire for Children [17], between-group Cohen *d* effect size=1.28), general anxiety (Beck Anxiety Inventory [18], *d*=1.47), and depression (Montgomery-Åsberg Depression Rating Scale [19], *d*=1.39) [13]. In another study, Spence et al [12] reported comparable, significant changes in clinician ratings of anxiety severity among adolescents with a markedly impairing anxiety disorder (predominantly GAD and social phobia) who completed 10 ICBT sessions (*P*<.001) and clinic-based, face-to-face delivery of CBT (*P*<.001); in contrast, those in a wait-list control condition displayed no significant change in severity [12]. In a recent single-group open trial published by Silfvernagel et al [11], a large within-group treatment effect emerged for adolescents with mild-to-moderate anxiety who completed 6 to 9 ICBT treatment modules (*d*=2.51). Most recently, Stjerneklar et al [14] reported that, compared with wait-list control, more adolescents who received ICBT were classified as being free of their primary anxiety disorder as well as any other anxiety disorder posttreatment (*P*<.05), with the odds of being free of their primary disorder appearing 3.60 times greater for recipients of ICBT. Participation in ICBT was also associated with greater improvements in clinician-rated diagnostic severity (*P*<.05) and adolescent- and mother-rated improvement in anxiety symptoms (*P*=.001). Many positive effects of ICBT were maintained at the 3-month follow-up, including freedom from anxiety diagnoses and subjective improvement in symptomatology. At this time, broad recommendations for future research within the ICBT field include conducting power calculations to ensure adequate sample sizes, defining primary outcomes before conducting the study, presenting results from intention-to-treat analyses, and measuring and reporting treatment adherence [20].

### Objectives

We conducted a pilot RCT to inform the planning of a definitive RCT to test the effectiveness of the ICBT program, Breathe (Being Real, Easing Anxiety: Tools Helping Electronically) compared with a static webpage listing anxiety resources (considered a form of usual self-led care during internet use). In the pilot RCT, we set out to (1) determine a sample size for the definitive RCT; (2) define a minimal clinically important difference (MCID), as defined by adolescents, for the primary outcome measure; (3) estimate recruitment and retention rates to determine the number of study sites needed and the timeline for recruitment; (4) measure intervention acceptability to inform critical intervention changes; (5) determine the use of cointerventions; and (6) conduct a cost-consequence analysis to inform a cost-effectiveness analysis for the definitive RCT.

## Methods

The study design was a two-arm pilot RCT (Breathe vs a static webpage) conducted with adolescents aged 13 to 17 years across Canada. We received approval from the Health Research Ethics Boards at the University of Alberta (Edmonton, Alberta), Izaak Walton Killam Health Centre (Halifax, Nova Scotia), and the Children’s Hospital of Eastern Ontario (Ottawa, Ontario) to conduct the study. The study protocol was registered with ClinicalTrials.gov (NCT02059226) and published [21].

### Recruitment

We recruited adolescents between April 2014 and May 2016 over the course of 3 recruitment cycles. Near the end of each cycle, we reviewed the effect of the different recruitment strategies that we employed. Cycle 1 recruitment spanned April 2014 to August 2015 and involved a *soft launch* with health care professionals providing study pamphlets to prospective participants seeking mental health care from emergency departments, mobile or school-based crisis teams, and primary care clinics in Edmonton, Alberta; Halifax, Nova Scotia; and Ottawa, Ontario. Cycle 2 spanned 12 months (September 2014 to September 2015) and involved the implementation of a communication strategy with health care providers and study site contacts. The communication strategy involved the distribution of study updates through email (using MailChimp) [22] on a monthly basis and fostering relationships between Breathe research staff and recruitment partners through teleconferences and site visits, as requested. In cycle 3 (October 2015 to May 2016), we introduced a social media recruitment strategy (Facebook, Twitter, and Instagram) with posts that appeared when adolescents searched or posted about anxiety or stress. These posts directed adolescents to the study website, which provided details about the study, instructions for eligibility screening and potential enrolment, information on anxiety disorders, and contact information for the research team.

### Screening for Study Eligibility

Youth eligible for participation were Canadian adolescents aged 13 to 17 years who (1) could read and write English, (2) had regular access to a telephone and a computer system with a high-speed internet service, (3) were able to use a computer to interact with web-based material, and (4) reported the presence of anxiety symptoms (Multimedia Appendix 1).

The exclusion criterion was adolescent self-report of suicidal thoughts in the past week. In cycle 1 recruitment, we initially had a second exclusion criterion, receipt of face-to-face CBT; however, we removed this criterion midway in the first recruitment cycle (cycle 1) due to emails from adolescents who found it confusing (eg, unaware of what CBT is, difficulty distinguishing CBT from other health care services such as support from a guidance counselor). Upon reviewing the questions we asked adolescents about their participation in other services that would result in ineligibility, we inferred that adolescents were seeking the Breathe program as an adjunct to other counseling and school-based services (not necessarily CBT-based) and that this would likely reflect how the program would be used in a real-world setting. Although we originally implemented this criterion as a way of reducing the potential for cointervention during the definitive trial, removing it increased the extent to which the planned definitive trial would evaluate real-world treatment effectiveness. At the time of this protocol change, 150 adolescents had been deemed ineligible for study enrolment due to this criterion.

We screened adolescents for study eligibility using a 2-stage process:

- Stage 1: During stage 1, we screened adolescents on inclusion criteria 1 to 3 and the second exclusion criterion until it was removed. Adolescents used a secure web-based process to answer questions to determine eligibility [23]. Telephone-based and email support during this stage were available from a research team member.
- Stage 2: Adolescents who met the first set of criteria proceeded to stage 2 screening. Stage 2 screening was conducted via the secure, internet-based platform, Intelligent Research Intervention Software (IRIS) [24]. During this stage, we assessed adolescents on inclusion criteria 4 and 5, and exclusion criterion 1.

We screened adolescents for anxiety symptoms using the Screen for Child Anxiety-Related Emotional Disorders (SCARED) [25].

To be eligible for study participation at this stage, SCARED scores needed to indicate the presence of anxiety symptoms. Adolescents were not excluded from study participation based on their SCARED scores. Although we originally thought that the Breathe program could be used by adolescents with mild-to-moderate anxiety symptoms [21], we did not exclude adolescents whose SCARED scores indicated severe anxiety symptoms. This approach in our pilot trial allowed us to determine who was accessing the program and identify the target population for the definitive trial.

We assessed the risk of deliberate self-harm using the 4-item Ask Suicide-Screening Questions (ASQ) [26]. Adolescents who responded *yes* to any of the questions received a safety telephone call from a research team member who evaluated intent/severity/immediacy of risk before deciding on the adolescent’s safety and ability to participate. Adolescents who indicated an immediate risk of self-harm by responding *yes* to question 3 on the ASQ (“In the past week, have you been having thoughts about killing yourself?”) were excluded from the trial and received brief telephone-based support from the research team member who encouraged the adolescent to seek mental health care appropriate to their level of need.

### Informed Consent/Assent

Adolescents aged 15 to 17 years were asked to consent to the study on their own behalf; adolescents aged 13 and 14 years were asked to assent to study participation. We also required parental consent for all adolescents aged 13 and 14 years, even if they were assessed as being able to consent. The intent was to have parents involved so that they could support their child with the enrolment process.

Consent/assent from eligible adolescents was indicated electronically via the secure myStudies website [23]. The first webpage confirmed that the individual understood that he/she could ask questions about the study at any time during the study or in the future. Each webpage included a *Contact Us* button that provided a pop-up email box with toll-free phone and email contact information for a member of the research team. The *Contact Us* button triggered a message to the participant that a research team member would contact them to answer any questions they may have before proceeding with consent/assent. Individuals were also given the option of saving or printing a blank copy of the informed consent form to read and review on their own instead of proceeding immediately to consent at that time. During the consent/assent process, individuals were guided through a series of sections describing what it meant to participate (eg, reiterating the youth’s right to withdraw from the study at any time) and asking them to confirm (through true/false and yes/no questions) that they understood the study, its risks and benefits, and/or had any questions. True/False questions were added before the final consent/assent webpage to ensure that the individual understood the study information. If an individual answered incorrectly, a pop-up box with the correct answer and explanation appeared. The script was designed to give individuals ample time to make an informed decision about participation. Once consent/assent was indicated, the date and time of consent/assent were recorded by the myStudies website [23].

### Randomization and Blinding

Randomization took place after informed consent/assent was obtained. Adolescents were randomly assigned using a computer-generated allocation sequence with a 1:1 ratio to 1 of 2 groups. A graduate student trainee affiliated with the project generated this sequence and an email was sent to each participant with information on their assigned intervention and log-in/website information to begin participation. A permuted block randomization procedure [27] with random block sizes of 4 to 6 was used. Given the methodological objectives of the pilot study, no blinding took place.

### Experimental Group: The Breathe Program

#### Program Details

Consistent with published treatment recommendations [7], the Breathe program is a newly developed 8-module CBT program that involves: (1) multimedia-based education about anxiety problems and approaches to overcoming anxiety (eg, reviewing why exposure exercises are important); (2) self-assessment activities to determine level of treatment and safety needs; (3) activities that teach users about anxiety sensitivity, how to identify anxious thoughts, and how to develop realistic thinking about anxiety-producing situations; (4) activities for practicing coping and relaxation skills with self-assessment of performance and rewards; (5) development of a hierarchy of feared situations and steps for gradual and repeated exposure to feared situations (using imagery and *in vivo* activities); (6) contingency management (examining the function of anxiety from a reinforcement perspective) and modeling (viewing videos of others confronting feared situations); and (7) skills for maintenance and relapse prevention. An overview of module content is provided in Table 1.

**Figure 1:**
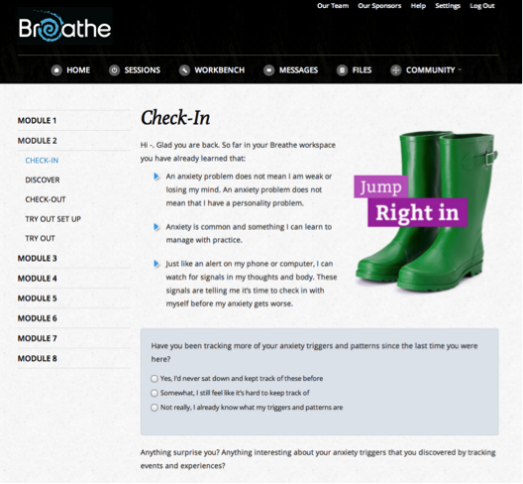
Being Real, Easing Anxiety: Tools Helping Electronically check-in activity.

Animations, embedded video, audio playback, graphic novel style vignettes, image maps, timed prompts, and on-screen pop-ups were used in each module to provide an interactive and multimodal experience. Each module included 4 components: *Check-in*, which asked the youth to assess and rate their social-emotional functioning over the past week (Figure 1); *Discover*, which introduced the module’s key topics; *Check-out*, which asked the youth to reflect on their responses to module content; and *Try Out*, which outlined activities for the adolescent to choose to practice the module’s key concepts and skills. Check-in/Check-out ratings that indicated thoughts of self-harm triggered a safety video and pop-up box, encouraging the adolescent to notify a parent/guardian of their thoughts and to seek immediate help. For each module, adolescents were given a choice as to whether they wanted parents to receive an email that included educational materials about the nature of adolescent anxiety and highlights of key topics that they worked on for that module. During program use, adolescents were also provided with the email contact of a trained research team member who could answer questions about the program and/or treatment (including discussion of distressing issues that may be activated during treatment). The program underwent an evaluation for usability with adolescents and clinicians before the start of the trial to improve the intervention’s technical interface, therapeutic messaging, and user experience (eg, esthetics, presentation of rating scales) [28].

**Table 1:** Overview of Being Real, Easing Anxiety: Tools Helping Electronically (Breathe) program content.

Module	Content overview
Module 1	Psychoeducation Introduction to Breathe and the topic of anxiety (eg, fight or flight response, normalization of anxiety)
Module 2	Realistic thinking Introduction to unrealistic beliefs and their role in anxiety Strategies for catching, challenging, and changing unrealistic beliefs
Module 3	Cognitive distortions Overview of relationship between thoughts, feelings, and behavior Introduction to common thinking traps that fuel anxiety
Module 4	Relaxation skills Introduction to/practice with relaxation strategies (deep breathing, visualization, and progressive muscle relaxation)
Module 5	Avoiding avoidance Introduction to the role of behavior (particularly avoidance) in fueling anxiety Creation of a rewards list for taking steps toward facing anxiety
Module 6	Constructing a fear hierarchy Instructions for creating a fear hierarchy, including examples of hierarchies Creation of a fear hierarchy
Module 7	Fear hierarchy practice Introduction to strategies for completing exposures and facing fears (eg, video examples of other youth working on fear hierarchies and visualization activities)
Module 8	Concept integration Reinforcement of links between strategies for identifying/challenging unrealistic beliefs, negative thinking, and behavioral changes Strategies for addressing challenges that often accompany anxiety (eg, social skills and body image)

#### Persuasive Design Mechanisms

**Figure 2:**
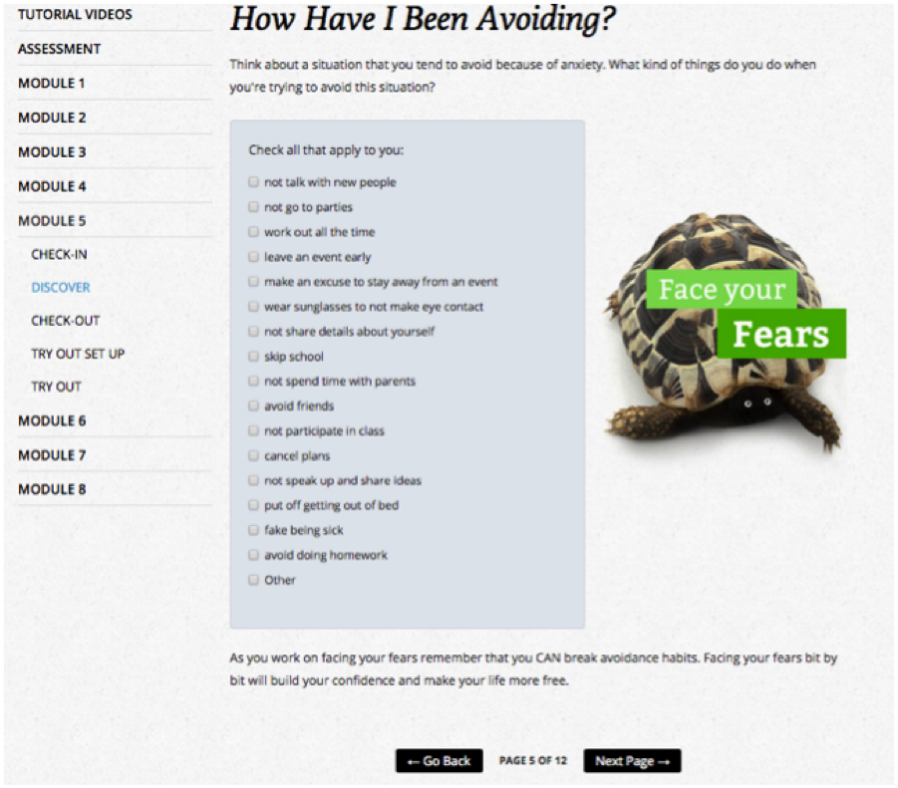
Example of adolescent-provided personal information for module tailoring.

Breathe was delivered via IRIS, the same platform used for eligibility screening. The platform supports the integration of persuasive design [29] and enables a personalized program experience for adolescents via 3 primary mechanisms: (1) *tailoring content*, which involved adolescents providing information for the program to use during the module (Figure 2); (2) *self-monitoring* that enabled the adolescent to track their own behavior toward intended outcomes; and (3) *automated reminders*—for example, adolescents were instructed at the beginning of the trial to use the program weekly and those who did not log in for 1 week received an email encouraging them to complete their weekly module.

### Control Group: Static Webpage

Adolescents assigned to the control group received minimal intervention—access to a secure, password-protected static study webpage housed in IRIS. The website offered suggested anxiety-related trade publications, print-based workbooks for adolescents, and the names of national and local organizations and websites where the adolescent might find support. There was no interactivity or personalization included in the webpage. Adolescents assigned to the control group were provided with the option to access the Breathe program for clinical use at the end of their 8-week control group participation.

### Safety Monitoring

Among those adolescents allocated to the Breathe program, a graduate student trainee monitored adolescent well-being under the supervision of a child and adolescent psychiatrist and the primary investigator. Adolescent well-being was monitored via adolescents’ answers in the check-in and check-out components of the program. Automated indicators built into the IRIS program flagged safety issues (eg, decompensation in anxiety symptoms, thoughts of self-harm), and an email notification was sent to the trainee. The trainee would then (1) develop a plan of action and discuss it with the psychiatrist and principal investigator and (2) subsequently contact the adolescent and their parent(s) by phone follow-up within 36 hours. Serious adverse events were to be reported to the institutional ethics board. Among adolescents randomized to the control group, the website provided contact information for local emergency resources (crisis lines, emergency department, and/or other crisis mental health resources).

### Study Procedures

Study participation did not begin until the adolescent logged into their assigned intervention via IRIS. Once logged in, participants could access either the Breathe website or the static webpage and could complete the study outcome measures. Outcome measures were available to complete at baseline (available immediately upon first log-in; preintervention), after the completion of module 8 (postintervention; experimental group) or 8 weeks of website access (postintervention; control group), and at 3 months postintervention (follow-up).

### Data Collection

#### During Eligibility Screening

We used the SCARED [25] to screen interested adolescents for anxiety symptoms. The SCARED is a 41-item self-report screen for symptoms of panic disorder, social anxiety disorder, and GAD in clinical and community adolescent samples as based on diagnostic criteria [30-32]. Adolescents responded to the 41 items of anxiety symptoms/experiences as *not/hardly ever true*, *somewhat/sometimes true*, or *very/often true*.

#### During Study Participation

##### Study Recruitment and Retention

A study log was used by a research coordinator to track the number of adolescents who were screened as eligible and were enrolled or not enrolled in the study as well as those who completed outcome measures at postintervention and 3-month follow-up.

##### Demographic Characteristics

Self-reported age, gender, and province of residence, as well as adolescent and parent contact information (ie, telephone number and email), were collected from enrolled adolescents via IRIS before starting the intervention.

##### Anxiety Symptoms

We used the total score from the Multidimensional Anxiety Scale for Children–Second Edition (MASC2; 50 items) to measure anxiety symptoms pre- and postintervention. The MASC2 is one of the most widely used self-report measures in clinical trials in adolescents with anxiety disorders. It assesses physical symptoms, social anxiety, harm avoidance, separation/panic, and total anxiety, and has excellent 3-month test-retest reliability [33] and validity [34,35]. MASC2 software scores adolescent responses, produces a total raw score, and converts raw scores to T scores (a standardized score that allows for individual scores on a dimension to be compared with those from a broader population in which the dimension is normally distributed). On the MASC2, converting raw scores into T scores allows for anxiety scores to be differentiated as average/typical (scores between 45 and 55), slightly above average (scores between 56 and 60), above average (scores between 61 and 65), much above average (scores between 66 and 70), and clinical diagnosis (score>70) [33].

We administered the MASC2 to adolescents in the experimental group at baseline and at each posttreatment time point. We used the data collected at the 3-month time point to estimate data completion rates for the full-scale trial. Adolescents in the control group completed the MASC2 at baseline and 8 weeks after study enrolment.

##### Minimal Clinically Important Difference

The MCID was defined using data collected from adolescents allocated to the experimental group following module 8 completion (posttreatment). Adolescents were asked to indicate the minimum change in anxiety for which they would consider it worthwhile to participate in the Breathe program. We used adolescents’ self-reported global ratings of change on a 10-point Likert scale (−5=*a very great deal worse* to +5=*a very great deal better*), a commonly used anchor [36,37]. We did not collect data from adolescents who completed the Breathe program after their 8-week period in the control group.

##### Program Acceptability

Adolescents allocated to the Breathe program answered 16 questions on program acceptability after completing module 8. An instrument was designed specifically for this study to assess ease of program use, sense of privacy, and delivery format and content. The research team reviewed the instrument for face and content validity. For 10 questions, a 4-point Likert scale (*strongly disagree* to *strongly agree*) was used. We originally intended to use a 5-point scale but removed a *neutral* option on the scale to improve the interpretability of adolescents’ ratings. Scores ranged from 10 to 40, with higher scores indicating higher acceptability. Of the remaining 6 questions, one asked adolescents to identify topics they would like to see in future Breathe programs by selecting from a list of options (eg, bullying, specific phobias). Two questions allowed adolescents to identify the top 3 most motivating program features and 3 most helpful modules from provided lists. Three questions were open-ended, allowing adolescents to note challenges or barriers that they faced in taking part in the trial, the extent to which they used the skills learned, and any technical issues encountered while completing the program.

We assessed treatment adherence to further evaluate program acceptability. Adherence was measured by documenting the number of modules completed by adolescents allocated to the Breathe program. We also recorded whether adolescents allocated to the control intervention accessed the website during the 8-week assignment period. Adherence data were recorded by and stored in IRIS.

##### Health Care Resource Use

We asked adolescents allocated to Breathe to report on the nature and frequency of health care use (cointerventions, emergency department visits, other treatments, and medication) during completion of the program. This information was collected following module 8 completion. We also detailed software development and maintenance costs (for Breathe program maintenance and delivery) and any training and personnel costs associated with the Breathe program.

### Outcomes

The primary outcome was self-reported change in anxiety symptoms from baseline to 8 weeks posttreatment. Given the purpose of this pilot trial, primary outcome data were not used to estimate treatment effects. Rather, the outcome data were described and used to inform the sample size needed for a definitive trial. Secondary outcomes were study recruitment and retention rates, MCID, program acceptability, and health care resource use during the trial.

### Sample Size

The sample size calculation for the pilot RCT was based on obtaining data to determine the sample size necessary for a definitive RCT [38,39]. We set out to enroll 80 adolescents (40 assigned to each group) and expected to retain 40 adolescents (20 per group) at the 8-week time point. We estimated that we would need 20 adolescents per group at the 8-week time point to estimate SDs and provide 80% CIs for SDs and 95% CIs for recruitment and retention proportions with sufficient levels of precision in our calculations.

### Analysis

#### Primary Outcome

For each group, we calculated the mean difference in raw MASC2 scores (and SDs) from baseline to 8 weeks (postintervention). Per protocol, we used data from adolescents who had completed the MASC2 both pre- and postintervention for this analysis. A two-sided two-sample *t* test power analysis was conducted for the change in score from baseline to postintervention to calculate the sample required per group in a definitive RCT [40]. Given the various effect sizes based on different comparators and the heterogeneity between previous studies [20], we were conservative and decided that we wanted to be able to detect a medium treatment effect (*d*=0.3) of the intervention on our primary outcome for the experimental group in the definitive RCT. We used the software R for the sample calculation (type I error=0.05; power=0.80).

#### Secondary Outcomes

We used descriptive statistics (eg, mean and frequency) to summarize demographic characteristics, recruitment and retention rates, health care utilization, and program acceptability. Participant data were considered *unknown* if no answer was recorded. We used SPSS version 24 for all secondary analyses [41].

We defined the recruitment rate as the number of adolescents enrolled during the study period divided by the number of adolescents eligible to participate during the study period. The recruitment rate was iteratively calculated throughout the trial to assess the adequacy of the recruitment strategy and formally determined at 26 months (the conclusion of the study recruitment/enrolment period) to determine an overall timeline for the definitive trial. Retention rates were defined as the number of adolescents who completed outcome measures at the 8-week (posttreatment retention) and 3-month (follow-up) time points divided by the number of adolescents enrolled. We used the 8-week retention rate to adjust the sample size for the definitive trial (eg, to adjust for anticipated study attrition).

We intended to use adolescent global ratings of change (within the ranges of +2 to +3 or −3 to −2 for reported change using a 10-point Likert scale) reported at the end of Breathe program completion to estimate the MCID value [42]. However, only 13 of the 36 adolescents who received the Breathe program reported data for this outcome, and we considered the data set inadequate to support the analysis. Instead, we report the global ratings of change for these participants with no calculation of the MCID. We will carry over the MCID objective to the definitive trial with a larger sample size.

Analysis of program acceptability data included summarizing the number of modules completed, and examining whether there were differences (eg, by gender) between program completers (>75% of modules) and noncompleters to identify potential confounders that would need to be adjusted for in the definitive RCT. We summarized responses to the satisfaction instrument using mean and frequencies and collated answers to open-ended questions. Completers and noncompleters were compared using two-sided two-sample *t* tests for continuous data and chi-square tests of independence for categorical data. A *P* value less than .05 was considered to be statistically significant.

We intended for health care resource use data to inform a preliminary cost-consequence analysis [43,44]. However, data for this outcome were largely missing, and the data set was not adequate to support this analysis. Instead, we report the type and frequency of health care use and the crude costs associated with the Breathe program. We will investigate the cost-consequence objective in the definitive trial with a larger sample size.

## Results

### Recruitment and Retention Rates

Recruitment commenced in April 2014 and continued until the end of May 2016 for a 26-month recruitment period. During this time, 588 adolescents were screened for study eligibility. A total of 94 adolescents were confirmed eligible for study participation (94/588, 15.9% of those screened; 95% CI 13.2% to 19.3%); all consented and were enrolled in the study. The success rates of the 3 recruitment cycles are presented in Table 2. The most dramatic increase in recruitment was observed following the introduction of social media to the recruitment strategy with an approximate increase of 300% in enrolled adolescents when comparing cycles 1 and 2 (29 months combined) with cycle 3 (8 months).

**Figure 3:**
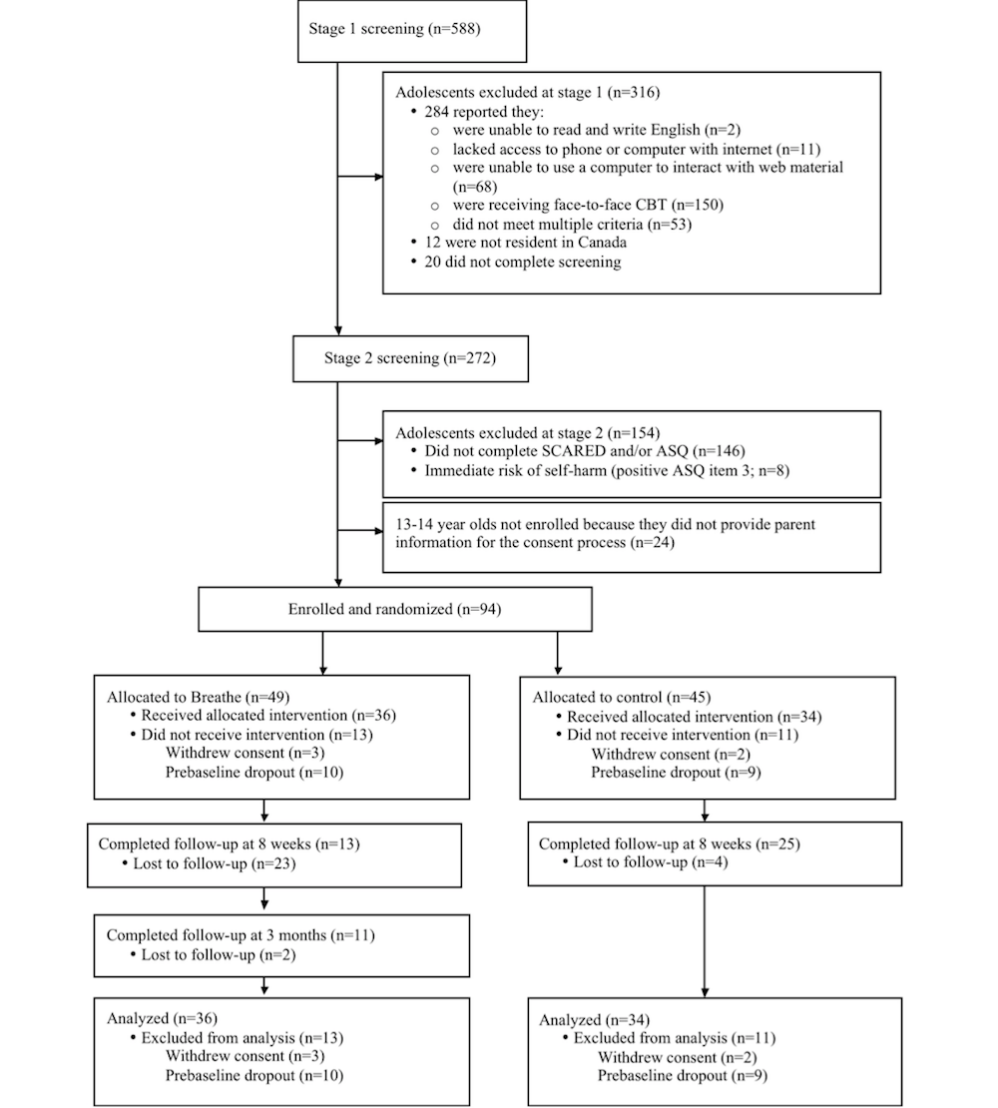
Consolidated Standards of Reporting Trials diagram describing flow of participants through the study. ASQ: Ask Suicide-Screening Questions; CBT: cognitive behavioral therapy; SCARED: Screen for Child Anxiety-Related Emotional Disorders.

The flow of participants through the trial is shown in Figure 3. We could not confirm the eligibility of 146 adolescents at stage 2 screening as they did not complete screening measures: 111 did not complete the SCARED and the ASQ, and 35 completed the SCARED but not the ASQ. Thus, we enrolled 94 of 240 potentially eligible adolescents (39.2% recruitment rate). A total of 49 adolescents were allocated to the Breathe program, and of these adolescents, 36 accessed the program; 45 adolescents were allocated to the control intervention, and of these adolescents, 34 accessed this intervention. The study’s retention rate at 8 weeks was 28% (13/46; 95% CI 17%-44%) for the Breathe intervention group and 58% (25/43; 95% CI 42%-73%) for the control group. The overall 8-week retention rate (both groups combined) was 43% (38/89; 95% CI 32%-54%). The retention rate at 3 months among adolescents allocated to the Breathe intervention group was 24% (11/46). The analysis was based on the 70 adolescents who completed baseline measures and did not withdraw from the study. No serious adverse events were detected during the study.

**Table 2:** Number of youth recruited in each recruitment cycle.

Group	Cycle 1, n (%)	Cycle 2, n (%)	Cycle 3, n (%)	Total across cycles, n (%)
Youth interested and screened	11 (1.9)	33 (5.6)	544 (92.5)	588 (100.0)
Youth enrolled	5 (5)	14 (15)	75 (80)	94 (100)

### Description of the Study Sample

Participant characteristics are shown in Table 3. The average age was 15.3 years (SD 1.2; range 13 to 17 years), and the majority of participants were female (63/70, 90%). On the basis of baseline MASC2 T scores, 7/70 (10%) adolescents reported average levels of anxiety, 3/70 (4%) reported slightly above-average levels, 5/70 (7%) reported above-average levels, 7/70 (10%) reported much above-average levels, and 46/70 (66%) were at a level consistent with a clinical diagnosis of anxiety.

**Table 3:** Baseline characteristics of participants by study group.

Characteristics		Total (N=70)	Breathe^a^ intervention group (n=36)	Control group (n=34)
**Age (years)**				
	Mean (SD)	15.3 (1.2)	15.6 (1.1)	15.1 (1.4)
	No response, n (%)	2 (3)	1 (3)	1 (3)
**Gender, n (%)**				
	Female	63 (90)	34 (94)	29 (85)
	Male	6 (9)	2 (6)	4 (12)
	No response	1 (1)	0 (0)	1 (3)
**Geographic region in Canada, n (%)** ^b^				
	West Coast	6 (9)	2 (6)	4 (12)
	Prairies	36 (51)	18 (50)	18 (53)
	Central	22 (31)	13 (36)	9 (27)
	Atlantic	6 (9)	3 (8)	3 (9)
**MASC2** ^c^ **T score**				
	Mean (SD)	71.4 (12.0)	71.7 (13.3)	71.2 (10.6)
	IQR^d^ (Q1, Q3)	14 (66.0, 80.0)	22 (62.0, 83.0)	11 (68.0, 78.0)
	No response, n (%)	0 (0)	0 (0)	0 (0)

^a^Breathe: Being Real, Easing Anxiety: Tools Helping Electronically.

^b^The West Coast region includes British Columbia; the Prairies region includes Alberta, Manitoba, and Saskatchewan; the Central region includes Ontario and Quebec; the Atlantic region includes Nova Scotia, Newfoundland, New Brunswick, and Prince Edward Island.

^c^MASC2: Multidimensional Anxiety Scale for Children–Second Edition.

^d^IQRs are reported as MASC2 scores were not normally distributed as identified by Shapiro-Wilk tests (*P*<.05).

### Anxiety Change Scores and Sample Size for a Definitive Randomized Control Trial

A total of 38 participants completed the MASC2 measure at both the baseline and the 8-week posttreatment time points (13/36 in the Breathe intervention group and 25/34 in the control group). Among adolescents in the experimental group, the mean change in raw MASC2 scores from 8-weeks posttreatment to baseline was −7.9 (SD 15.7). The 80% CI for the SD generated for the 8-weeks posttreatment to baseline change score was 12.6 to 21.7. For the control group, the mean change in MASC2 scores from 8-weeks posttreatment to baseline was −9.0 (SD 15.4). The 80% CI for the SD generated for the 8-weeks posttreatment to baseline change score was 13.1 to 19.1. Assuming 80% power and 5% type I error rate, 177 adolescents per group (354 total) will be able to detect an effect size of 0.3 using a two-sided two-sample *t* test for means. The pilot data suggest that the pooled SD could be 15.7, translating the effect size of 0.3 to a detectable difference of 4.7 in change scores between the Breathe group and the control group.

### Global Ratings of Change

Of the 13 adolescents who rated their change in anxiety symptoms after completing the Breathe program, 6 reported a *somewhat better* change and 7 reported a *much better* change.

### Program Acceptability

**Figure 4:**
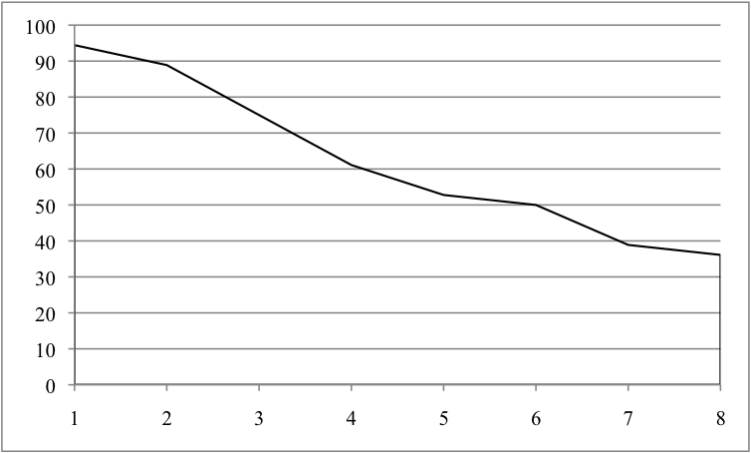
Percentage of adolescents who completed each Being Real, Easing Anxiety: Tools Helping Electronically module.

Of the 36 adolescents who received the Breathe program, 13 (36%) completed all 8 modules (Figure 4) and 2 (6%) did not complete any modules. Program completers and noncompleters did not differ significantly in their responses to any of the 4 ASQ screening questions (*P*=.32, .93, .49, and .49), the manner in which they learned about the study (social media/on the web, health care provider/guidance counselor, friend, or not specified; *P*=.17), age (*P*=.85), or baseline MASC2 T scores (*P*=.44). Completers and noncompleters could not be compared on self-identified gender due to the limited number of males enrolled in the study.

In total, 39% (14/36) of adolescents provided feedback on the Breathe program (Table 4). The mean satisfaction score among these adolescents was 28.5/40 (SD 4.0), indicating modest satisfaction. All but 1 adolescent indicated that the Breathe program was easy to use and that they understood all the material presented within the program; 36% (5/14) of participants noted that it was difficult to complete the Try Out (homework) pages each week. All participants liked that the program was completed on the web, with 79% (11/14) indicating no concerns with privacy. Responses were divided as to whether the program should include a social media component (5/14 in agreement), be more personalized to the participant (7/14 in agreement), and include a module for parents (8/14 in agreement). The most common barriers to program completion were difficulty completing exposure activities and remembering/finding time to complete modules, among other life commitments. Additional feedback provided by Breathe users is provided in the Multimedia Appendices 2-Multimedia Appendices 4.

**Table 4:** Adolescent feedback on the Being Real, Easing Anxiety: Tools Helping Electronically (Breathe) program.

Item	Adolescent rating			
	Strongly disagreed, n(%)	Disagreed, n (%)	Agreed, n (%)	Strongly agreed, n(%)
The Breathe program was easy to use.	0 (0)	1 (7)	7 (50)	6 (43)
I understood all the material/content outlined in the Breathe program.	0 (0)	1 (7)	10 (71)	3 (21)
I had concerns regarding my privacy while completing the Breathe program.	6 (43)	5 (36)	3 (21)	0 (0)
I liked that the Breathe program was completed online.	0 (0)	0 (0)	7 (50)	7 (50)
The Try Out pages were hard to complete each week.	2 (14)	7 (50)	5 (36)	0 (0)
The email reminders sent to me by the Breathe program were helpful.	4 (29)	7 (50)	2 (14)	1 (7)
The length of the modules in the Breathe program was too long.	4 (29)	10 (71)	0 (0)	0 (0)
The Breathe program should have a social media component.	3 (21)	6 (43)	4 (29)	1 (7)
I would like the Breathe program to be more personalized to me.	2 (14)	5 (36)	4 (29)	3 (21)
I think my mom/dad/guardian should have had their own parent modules in the Breathe program.	5 (36)	1 (7)	5 (36)	3 (21)

### Health Care Resource Use

In terms of costs to develop the Breathe program, software development totaled Can $51,405 (US $36,462), whereas personnel costs associated with program development and maintenance during the study (eg, technician and programmer costs) totaled Can $73,172 (US $51,911).

With regard to health care resource use outside Breathe, 39% (14/36) of adolescents in the study answered questions about health care resource use during the Breathe program. We summarize the findings from these adolescents, but acknowledge that the results may not reflect all adolescents allocated to the Breathe program. Counsellors (of unspecified professional background, designation, or theoretical orientation) were the most commonly used resources; 50% (7/14) respondents reported having seen a counselor for anxiety at least once during their participation in Breathe, with 5 having attended 5 or fewer visits and 2 having attended 9 or more. A total of 43% (6/14) respondents had visited family physicians for anxiety-related concerns, although these visits occurred only once for all but one of this group. Other resources were accessed relatively infrequently, with the exception of 1 adolescent who accessed social work 7 times over the course of Breathe (Table 5). During the trial, no adolescents were identified as requiring additional support based on their responses to survey questions completed before and after each module.

**Table 5:** Health care resource use reported by adolescents after completion of module 8 of the Being Real, Easing Anxiety: Tools Helping Electronically program.

Resource used	Response^a^		Frequency of use for accessors	
	No, n (%)	Yes, n (%)		
Medication	13 (93)	1 (7)		100%
Counsellor	7 (50)	7 (50)		1 visit (14%), 2 visits (14%), 3 visits (29%), 5 visits (14%), 9+ visits (29%)
Psychologist	11 (79)	3 (21)		3 visits (67%), 5 visits (33%)
Psychiatrist	12 (86)	2 (14)		3 visits (100%)
Social worker	12 (86)	2 (14)		2 visits (50%), 7 visits (50%)
Family physician	8 (57)	6 (43)		1 visit (83%), 2 visits (17%)
Emergency department	11 (79)	3 (21)		1 visit (67%), 2 visits (33%)
Admitted to hospital	13 (93)	1 (7)		100%
Other treatment	12 (86)	2 (14)		Breathing and visualization exercises (50%), meditation and homeopathy (100%)

^a^The total is greater than 100%; adolescents could report more than one resource having been used.

## Discussion

### Principal Findings

This RCT piloted procedures and obtained data on acceptability to determine feasibility for a definitive RCT that would test the effectiveness of an ICBT program for adolescent anxiety. The 3 key lessons learned from conducting the pilot study and being applied to plan for the definitive trial are as follows: (1) adolescents did not use all the Breathe resources provided, and adjustments to the program were necessary to increase program completion in the definitive trial; (2) recruitment by social media was the most successful modality for recruiting adolescents into the study and should be the primary recruitment strategy in the definitive trial to ensure timely study completion; and (3) protocol adjustments are necessary to increase study retention at each measurement time point to improve outcome data collection.

Several adjustments were made to the Breathe program in an effort to support adolescents’ abilities to navigate and complete the program. First, we streamlined content and reduced the number of modules from 8 to 6. This decision was based on our observation of a leveling off of completion around sessions 5 to 6. To achieve this reduction, the flow of Breathe content was streamlined and focused, and content considered unessential or potentially overwhelming was removed (eg, numerous exercises highlighting the same concept). We also increased the use of video content in the 6 modules as this mode was described as inspiring by adolescents and created new first-person narrative videos to reinforce concepts and support adolescents in relating topics to their own lives. Second, we noted that participant feedback pointed to Breathe’s exposure component as a significant barrier to successful program completion. Although exposure is widely viewed as one of the most important components of therapy in terms of producing lasting change in anxiety symptoms, it does produce discomfort and may have contributed to the lower retention rate in the Breathe group as compared with the control intervention. We made 2 changes to exposure activities in the Breathe program. First, we adjusted the flow of our content so that exposures were first introduced in module 2 (not module 6, as originally designed); with this change, the concept could be introduced gradually and promote adolescents’ sense of positive change as a result of participating in the program. Second, we added telephone-based support from a coach to module 2. The coach will help the adolescent build an exposure activity plan tailored to their specific needs, clarify any confusion about how to set up an exposure activity, and address perceptions of self-efficacy in completing exposure activities. A recent systematic review suggests that this type of ICBT support (ie, human components) may boost ICBT adherence and engagement, particularly when delivered at critical points in a program that participants may find difficult or taxing [45].

In this pilot study, we enrolled 94 adolescents over a 26-month period (approximately 4 adolescents enrolled per month). This recruitment rate is not feasible for enrolling 354 adolescents (before attrition) in the definitive RCT. In testing different recruitment strategies, however, we learned that recruitment was most successful via social media (39/94, 42% enrolled). We were able to recruit approximately 4 times the number of adolescents in cycle 3 (n=75) once we launched our social media strategy, compared with cycles 1 and 2 during which we relied on health care providers. For the definitive trial, we plan to hire a communications specialist to assist with social media recruitment efforts. The communication specialist’s role will be governed by the following key objectives: (1) to create study awareness and inform the target audiences (parents, adolescents, and health care providers) about the Breathe program and study; (2) to increase traffic to the study website; and (3) to increase recruitment of study participants, including specifically targeting males. More broadly, our social media strategy will involve consideration of the functions of each communication strategy and any associated costs (eg, advertising), development of study-branded, tailored content, including that geared toward males (eg, sport performance stress) to increase their recruitment; an established approach for social media use (eg, frequency of posts, refreshing content), and a strategy to enhance user privacy (eg, use of marketing headlines so that personal disclosure is limited when social media content is viewed) and safety (eg, monitoring of web-based posts).

To improve study retention in the definitive trial, we will budget for financial tokens of appreciation in an effort to increase the response rate to study questionnaires (US $25 for completing posttreatment questionnaires; US $25 for completing follow-up questionnaires). We will also streamline the initial screening steps for consent and eligibility to reduce early dropout and will include those identifying as receiving CBT and other forms of treatment for anxiety. As other similar trials did not report such low retention rates nor offered incentives [8-11], we do not know what to expect in terms of the impact of this strategy on study retention, but hope that it will increase our retention to 75% at postintervention and 50% at the 3-month follow-up. Accounting for a 50% attrition rate at the 3-month follow-up, 708 adolescents would need to be enrolled in the definitive trial to achieve our desired sample size.

Another important aspect of this pilot trial was our intent to define an MCID for adolescent anxiety. However, challenges with retention did not permit us to calculate an MCID as planned. A critical aspect of all ICBT programs is the degree of improvement adolescent users experience as a result of their use. The use of MCID estimates could help adolescents, parents, and health care providers select among ICBT programs with different effects and anticipate the meaningfulness of the expected differences in their effects (ie, their clinical significance). Moving forward, within the broader literature, there is no minimum sample size necessary for calculating an MCID for a patient-reported outcome for adolescent anxiety. However, studies exploring the use of MCID scores in intervention-based research noted that this value has been calculated and used meaningfully in studies with sample sizes of a minimum of approximately 60 participants [46]. Should our efforts to improve study retention in the definitive trial be successful, we will have sufficient data to calculate an MCID. This MCID will be used to support the interpretation of results from this trial as opposed to defining a sufficient sample size (to calculate statistical significance) as originally planned.

### Limitations

The most significant limitation of this pilot RCT was the lack of data at posttreatment and 3-month follow-up. Unlike in the pilot, where posttreatment and 3-month follow-up MASCs were only administered if/when participants completed all program modules, administration of follow-up questionnaires in the definitive RCT will occur independently of program progress—ideally increasing the data available at both follow-up points and limiting the extent to which it is subject to selection bias. Another important limitation was our reliance on adolescents’ own recall when providing information about their utilization of other health care services. Addressing this limitation is challenging, given the privacy considerations and logistics that would be associated with verifying self-report data with information from other sources. However, as discussed previously, support from research staff will be made available to participants throughout their involvement in the full-scale trial, and they will be encouraged to contact staff with any questions that may arise as they provide this information.

A final limitation was the exclusion of 150 adolescents early on in the trial due to their report of CBT participation. Although we do not know how many of these adolescents would have consented/assented to participate in Breathe, their exclusion introduces the potential for further selection bias in the study. In this study, initial recruitment in cycles 1 and 2 was also very slow until a social media component was included in cycle 3. That these changes occurred during the pilot study and not the definitive trial is important, and we do not anticipate needing to adjust this inclusion/exclusion criterion or the recruitment modality utilizing social media further. In the definitive trial, efforts will also be focused on increasing the number of male participants, which was a limitation to our pilot trial’s study population.

### Conclusions

This study aimed to determine the feasibility of a definitive RCT exploring the effectiveness of Breathe, an ICBT program for adolescents reporting anxiety, by piloting procedures and assessing intervention acceptability. Adolescents enrolled in Breathe reported modest satisfaction with the program, with most indicating that it had been easy to use and was readily understood. Still, adjustments to the program are required to reduce attrition and address the barriers that adolescents encounter when attempting to complete key program elements. These adjustments, including streamlining program access and content and providing phone coaching support for adolescents around challenging elements of the program, will ideally increase recruitment and retention within the study, thereby promoting timely completion of the Breathe program and supporting the completeness of our dataset at each outcome time point. Ultimately, with these adjustments, the definitive RCT will allow for a more in-depth exploration of the impact of the Breathe program on adolescents with anxiety and inform ICBT utilization within mental health care systems.

## Appendix

Multimedia Appendix 1 Trial inclusion and exclusion criteria.

Multimedia Appendix 2 Adolescents’ ratings of most inspiring Being Real, Easing Anxiety: Tools Helping Electronically features.

Multimedia Appendix 3 Adolescents’ ratings of most helpful Being Real, Easing Anxiety: Tools Helping Electronically modules.

Multimedia Appendix 4 Open-ended responses from adolescents on the extent to which they used the skills learned and topics to include in the Being Real, Easing Anxiety: Tools Helping Electronically program.

## Abbreviations

ASQ: Ask Suicide-Screening Questions
Breathe: Being Real, Easing Anxiety: Tools Helping Electronically
CIHR: Canadian Institutes of Health Research
DALY: disability-adjusted life year
GAD: Generalized Anxiety Disorder
ICBT: internet-based cognitive behavioral therapy
IRIS: Intelligent Research Intervention Software
MASC2: Multidimensional Anxiety Scale for Children–Second Edition
MCID: minimal clinically important difference
RCT: randomized controlled trial
SCARED: Screen for Child Anxiety-Related Emotional Disorders
