# Supplementary figures and images for: An Internet-Based Cognitive Behavioral Program for Adolescents With Anxiety: Pilot Randomized Controlled Trial
Source: JMIR Ment Health. 2020 Jul 24;7(7):e13356. doi: 10.2196/13356 (PMC7414416; doi:10.2196/13356)

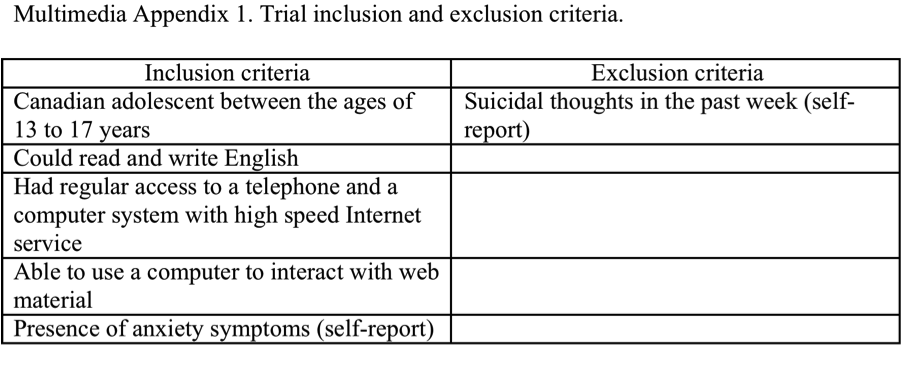

Supplement: Multimedia Appendix 1 [file mental_v7i7e13356_app1.png]

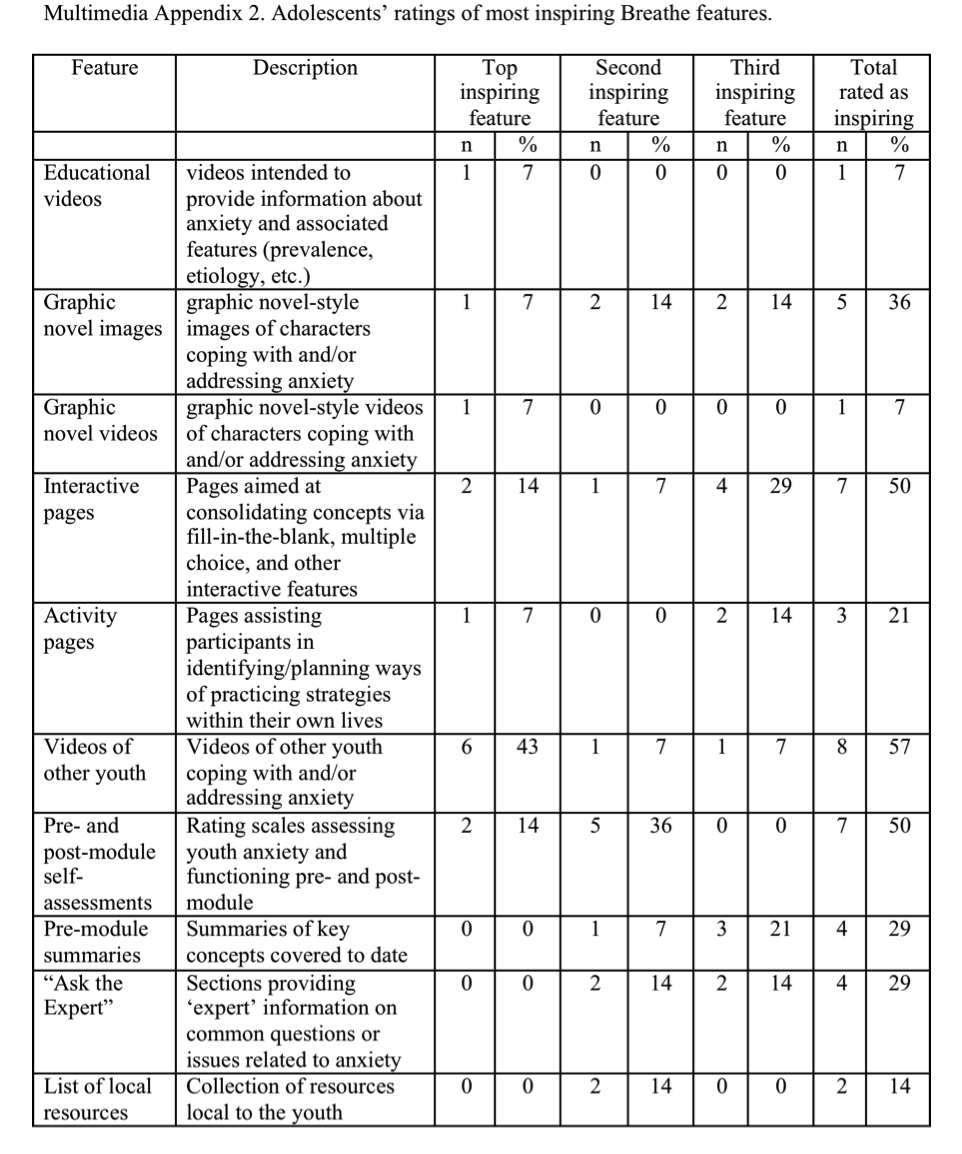

Supplement: Multimedia Appendix 2 [file mental_v7i7e13356_app2.png]

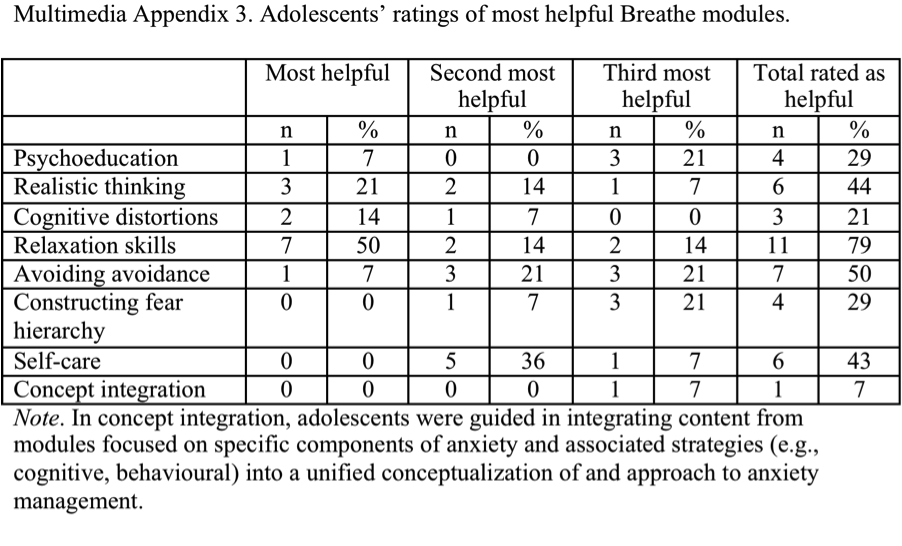

Supplement: Multimedia Appendix 3 [file mental_v7i7e13356_app3.png]

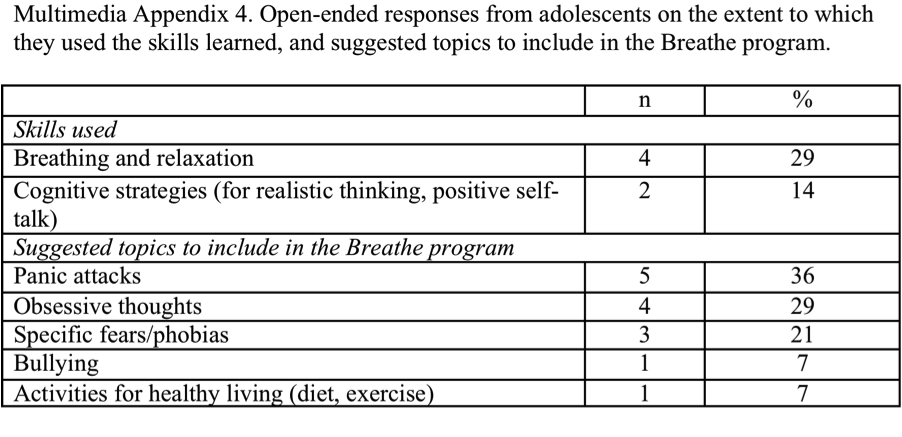

Supplement: Multimedia Appendix 4 [file mental_v7i7e13356_app4.png]
